# Survivin overexpression is potentially associated with pituitary adenoma invasiveness

**DOI:** 10.18632/oncotarget.22354

**Published:** 2017-11-10

**Authors:** Xiangyi Kong, Shun Gong, Lijuan Su, Xinqi Cheng, Honglei Li, Tingting You, Yanguo Kong

**Affiliations:** ^1^ Department of Neurosurgery, Peking Union Medical College Hospital, Chinese Academy of Medical Sciences, Beijing 100730, P.R. China; ^2^ Department of Breast Surgical Oncology, China National Cancer Center/Cancer Hospital, Chinese Academy of Medical Sciences and Peking Union Medical College, Chaoyang District, Panjiayuan, Beijing 100021, P.R. China; ^3^ Department of Neurosurgery, Shanghai Institute of Neurosurgery, PLA Institute of Neurosurgery, Shanghai Changzheng Hospital, Second Military Medical University, Shanghai 200003, P.R. China; ^4^ Department of Radiology, Brigham and Women's Hospital, Harvard Medical School, Boston, Massachusetts 02215, United States of America; ^5^ College of Computer Science and Technology, Zhejiang University, Hangzhou, Zhejiang 310027, P.R. China; ^6^ Department of Clinical Laboratory, Peking Union Medical College Hospital, Chinese Academy of Medical Sciences, Beijing 100730, P.R. China

**Keywords:** survivin, pituitary adenoma, invasiveness

## Abstract

**Background and objective:**

Survivin is an inhibitor of apoptosis. Its role in guiding the treatment of neoplasms, making diagnosis and predicting prognosis has been reported. However, there is little information on the implications and uses of survivin in predicting pituitary adenoma (PA) invasiveness. Existing information is unclear and controversial. We thus conducted this meta-analysis to explore whether the surviving expression levels in invasive PAs (IPA) and regular PAs are different or not. We considered both non-secreting and secreting tumors together.

**Methods:**

A global search strategy was systematically applied among five databases including Cochrane Library, Embase, PubMed, Web of Science, and Chinese National Knowledge Infrastructure (CNKI) up to June 18th, 2017. With a specially designed form including PAs’ invasive features, etc., data was collected. The included studies should present the data representing the surviving levels in IPA groups and regular PA groups, respectively. Differences were expressed as standard mean differences (SMDs) or odds ratios (ORs) with 95% confidence interval (CI). To estimate the heterogeneities, I^2^ test, Cochran's Q-test and Galbr figure were all conducted. A sensitivity-analysis and potential-publication bias were also performed.

**Results:**

In the present meta-analysis, 9 studies containing 489 patients were included. Seven studies with dichotomous-data showed that survivin over-expression in PA tissue was closely associated with a high invasive tendency (OR 6.226, 95% CI 3.970, 9.765; P<0.001), but 2 continuous-data studies revealed that there was no significant association (SMD −5.043, 95% CI-10.965, 0.878; p=0.095). A sensitivity-analysis suggested a statistically stable result. We did not find publication bias.

**Conclusion:**

We suggest that survivin overexpression is potentially associated with PA invasiveness. More research based on medical big data is needed to confirm this finding.

## INTRODUCTION

Pituitary adenomas (PAs) represent 10% to 25% of all intracranial neoplasms. PAs are generally divided into two categories based on their ability to invade the surrounding tissues; approximately 40% are invasive PAs (IPA) and most are being benign PAs [1]. Generally, IPAs are considered adenomas with proven growth to adjacent structures, like sphenoid sinus, bone and cavernous sinuses. The invasiveness can be observed through pre-surgical MRI, during surgery, or with histopathological analysis and demonstration of tumor spread to the bone, dura or nasal mucosa. The most common used method to identify invasion is sellar MRI and the Knosp grading system is also widely used [2]. However, the cellular morphological differences between these two types of PAs are mostly not significant, and it is difficult for neurosurgeons to distinguish between them using traditional pathological tests. Thus, precise diagnosis or treatment of IPA remains a challenge. The resection rate of IPA is low and the surgical risk, the mortality rate and the recurrence rate are high [3]. Therefore, it is necessary to explore new avenues such as available biomarkers.

Located on 17q25.3, survivin is considered to be an inhibitor of apoptosis [4]. Survivin level increased in many human tumors including lung cancer [5], breast cancer [6] and gliomas [7]. Some studies also confirmed that survivin is closely associated with PA invasiveness and tumorigenesis by blocking apoptosis via an effect on caspase-9, which is activated through extrinsic and intrinsic pathways [8, 9]. Survivin is thus considered to be a potential bio-marker for prognosis and a potential bio-target for treatment.

The values of survivin in PA invasiveness, however, is still unknown, and there have been conflicting reports [8, 9]. A few studies showed that a high survivin expression is associated with PA invasiveness [10], while Waligorska-Stachura et al. did not find any significant changes in the survivin level and in its splice variant transcripts in invasive and non-invasive PAs [8]. Many confounding factors may affect the study outcomes, such as follow-up, selected population, and study methods. Considering that a meta-analysis can solve between-study heterogeneities, we extracted all data from previous studies and systematically evaluated the essential implications of survivin in PA invasive features.

## RESULTS

### Search results and study characteristics

**Figure 1:**
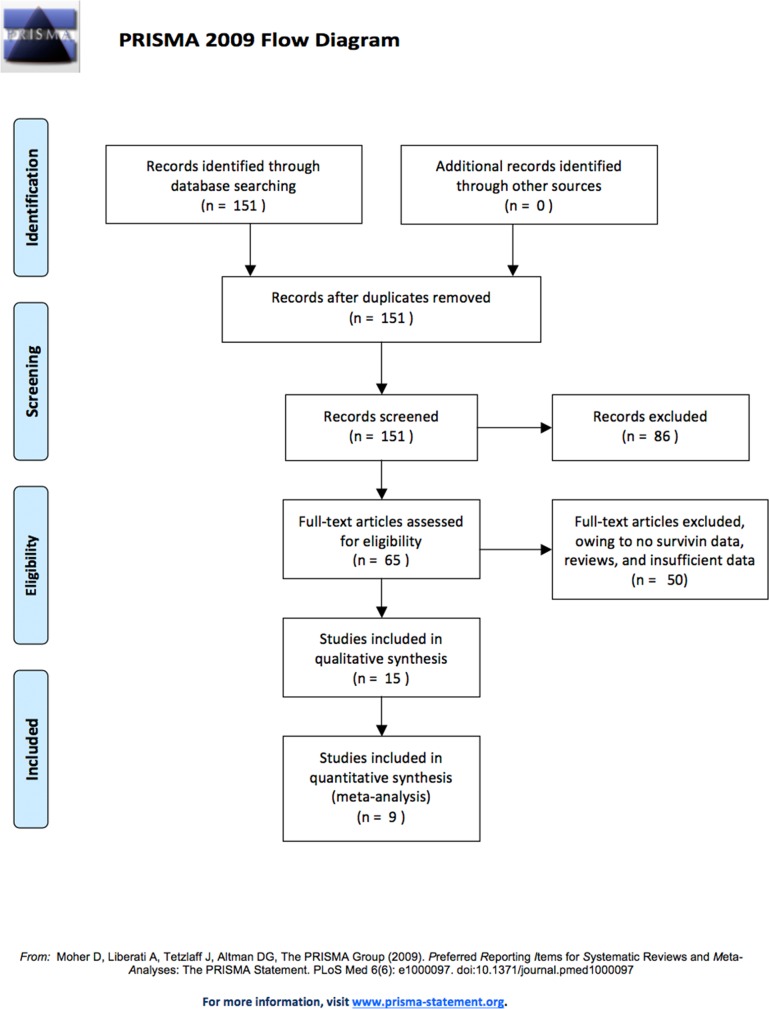
Literature search and selection of articles

According to PRISMA statement, a study selection flowchart was reported in Figure 1. A total of 151 studies were identified in search: 26 in Pubmed, 31 in Embase, 0 in Cochrane Library, 25 in Web of Science, and 33 in CNKI (Table 1). According to their title and abstract, 86 articles were excluded for the following reasons: 21 for no correlation with PA's invasiveness; 35 for no correlation with survivin; and 30 for *in vitro* and *in vivo* studies. The remaining 65 articles underwent further evaluation and screening; among these, 50 articles were excluded because they were reviews, or had insufficient data or no survivin data. Finally, a total of 9 articles met the inclusion criteria, eight of which were conducted in China and one in Poland. Table 2 was used to access the literature quality. Excluded studies and the rational for exclusion were listed in Table 3. The characteristics and methodological quality assessment results of the literatures included in this meta-analysis are shown in Table 4.

**Table 1:** Searching strategies and results for different databases

Database	Database URL	Search strategy	Results
**Pubmed**	https://www.ncbi.nlm.nih.gov/pubmed/	“pituitary neoplasms”[MeSH Terms] OR (“pituitary”[All Fields] AND “neoplasms”[All Fields]) OR “pituitary neoplasms”[All Fields] OR (“pituitary”[All Fields] AND “adenoma”[All Fields]) OR “pituitary adenoma”[All Fields]) AND survivin[All Fields]	26
**Embase**	https://www.embase.com/	‘pituitary’:ab, ti AND ‘survivin’:ab, ti	31
**Cochrane Library**	http://www.cochranelibrary.com/	survivin AND pituitary	0
**Web of Science**	http://apps.webofknowledge.com/	TOPIC: (survivin AND pituitary)Timespan: All years. Indexes: SCI-EXPANDED, SSCI, A&HCI, CPCI-S, CPCI-SSH, BKCI-S, BKCI-SSH, ESCI, CCR-EXPANDED, IC.	25
**CNKI**	http://www.cnki.net/	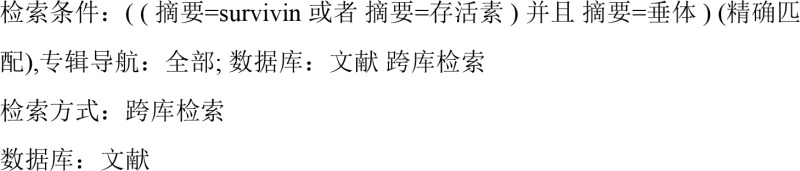	33

**Table 2:** Scale for methodological quality assessment

Criteria	Score
**1. Representativeness of cases**	
RA diagnosed according to acknowledged criteria.	2
Mentioned the diagnosed criteria but not specifically described.	1
Not Mentioned.	0
**2. Source of controls**	
Population or community based	3
Hospital-based RA-free controls	2
Healthy volunteers without total description	1
RA-free controls with related diseases	0.5
Not described	0
**3. Sample size**	
>300	2
200-300	1
<200	0
**4. Quality control of genotyping methods**	
Repetition of partial/total tested samples with a different method	2
Repetition of partial/total tested samples with the same method	1
Not described	0
**5. Hardy-Weinberg equilibrium (HWE)**	
Hardy-Weinberg equilibrium in control subjects	1
Hardy-Weinberg disequilibrium in control subjects	0

**Table 3:** Excluded studies and the rational for exclusion

Excluded studies	Rational for exclusions
Wasko et al. (2005)	This study only explored the survivin expression in different types of PAs
Zhang et al. (2008)	Not the most complete and the most recent article by these authors.
Jankowska et al. (2008)	Related data could not be abstracted from the results.
Wasko et al. (2009)	Comparison of survivin expressed between pituitary tumors and normal pituitary.
Formosa et al. (2012)	Comparison of survivin expressed between pituitary tumors and normal pituitary.
Waligórska-Stachura et al. (2015)	This study only explored the survivin and its splice variants ΔEx3 and 2βin different types of PA and non-cancerous pituitary tissues.
Dellal et al. (2015)	This study investigated serum survivin levels in patients with prolactinoma and demonstrate its value in diagnosis of the disease.
Stache et al. (2016)	This study investigated the underlying mechanisms responsible for high tumor recurrence rates of adamantinomatous craniopharyngioma, not related to PA.
Waligórska-Stachura et al. (2017)	This study investigated survivin and its splice variants DEx3 and 2B expressions in PAs and normal pituitary glands, not related to PA invasiveness.

**Table 4:** Characteristics and quality scores of the nine included studies

Year	Study ID	Ethnicity	Number	Mean age (year)	Male	Non-invasive	Invasive	Method	Survivin expression location	Positive (%)	Data type	Quality
2005	Shi QH et al.	Asian	71	42.5	33	32	39	IHC-SP	Nucleus or cytoplasm	43.66	Dichotomous	7
2006	Zhang ZQ et al.	Asian	48	35.8	26	21	27	IHC-SP and TUNEL	Nucleus or cytoplasm	66.67	Dichotomous	7
2008	Zhou J. et al.	Asian	49	43.0(M) 38.9(F)	23	20	29	IHC-SP	Not clear	53.06	Dichotomous	6
2008	Wang CL et al.	Asian	82	42	38	31	51	IHC-MaxVison^TM^	Nucleus or cytoplasm	67.07	Dichotomous	8
2008	He XL. et al.	Asian	43	47.5	21	28	15	IHC-SP	Nucleus or cytoplasm	37.21	Dichotomous	6
2008	Jankowska A et al.	Polonais	22	Not clear	Not clear	7	15	Quantitative RT-PCR	NA	NA	Continuous	9
2009	Xiang W et al.	Asian	50	39	34	20	30	IHC-SP	Cytoplasm	NA	Continuous	6
2009	Zhang YC et al.	Asian	66	68.1±2.5(M) 65.3±4.3(F)	36	27	39	IHC-SP	Not clear	69.70	Dichotomous	8
2010	Zhang YX et al.	Asian	58	42.6	30	37	21	IHC-SP	Nucleus or cytoplasm	37.93	Dichotomous	7

A total of 489 patients were included and 266 cases were of the invasive type. The percentage of positive survivin expression varied from 37.9% to 69.7%. Patients with positive survivin were further investigated using IHC (8 studies) more often than RT-PCR (1 study). If the nucleus or cytoplasm was stained, survivin expression was considered to be positive. Different studies use different cutoff values to distinguish between low and high survivin expression. The detailed standards to evaluate the intensity of survivin staining are shown in Table 5.

**Table 5:** Detailed standards to evaluate the intensity of survivin staining in the included studies

Year	Study ID	Standards to evaluate the intensity of Survivin staining
2005	Shi QH et al.	-, no expression or positive cells was 0%; +, light yellow or ≤10%; ++, brown yellow or 11%∼50%; +++, brown or 51%∼75%; ++++, dark brown or >75%
2006	Zhang ZQ et al.	+, positive cells <10%, or the staining degree was light yellow; +++, positive cells >60% and most positive cells presented as yellow or brong yellow; ++, between the above
2008	Zhou J et al.	-, no expression; +, <25%; ++, 25%∼50%; +++, >50%
2008	Wang CL et al.	-, no expression; +, positive cells <25%; ++, 25%∼50%; +++, >50%
2008	He XL et al.	-, positive cells <5%; +, >5%
2008	Jankowska A et al.	NA
2009	Xiang W et al.	Score:0, positive cells <5%; 1, 5%∼24%; 2, 25%∼49%;3, 50%∼74%; 4, ≥75%
2009	Zhang YC et al.	-, no expression or positive cells was 0%; +, light yellow or ≤10%; ++, brown yellow or 11%∼50%; +++, brown/dark brown or >50%
2010	Zhang YX et al.	Score A (positive cells):0, <5%; 1, 5%∼24%; 2, 25%∼49%; 3, 50%∼74%; 4, ≥75%Score B (staining degree of positive cells):0, no staining; 1, light yellow; 2, brown yellow; 3, dark brownA*B:Negative, ≤1; Positive, >1

### Meta-analysis of survivin and PA invasiveness

**Figure 2:**
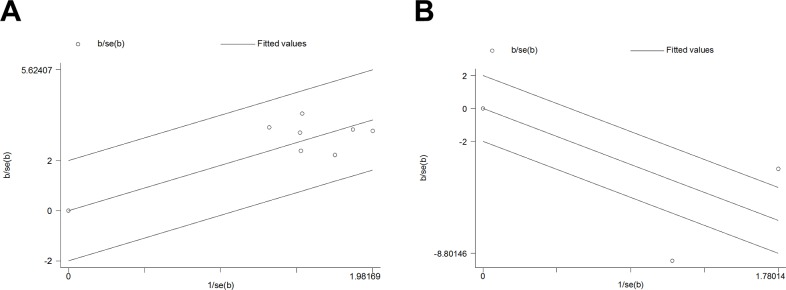
Galbraith figure of included studies focusing on the correlation between survivin and IPA **(A)** Seven studies with dichotomous data, and **(B)** Two studies with continuous data. If the circles are all distributed within the region bounded by the upper line and the lower line, this suggests that here is homogeneity. The farther away from the region, the more heterogeneity is present.

**Figure 3:**
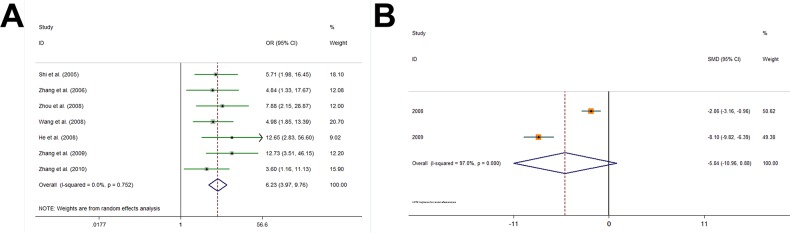
Individual and pooled effects of survivin and IPA **(A)** Using a random-effect model, seven studies with dichotomous data (OR 6.260, 95% CI 4.011, 9.770; P<0.001) showed an association between survivin and invasiveness. **(B)** Using a random-effect model, three studies with continuous data (SMD -5.043, 95% CI -10.965, 0.878; P = 0.095) also suggested that the association was statistically significant.

We divided PAs into the invasive and non-invasive types to merge the data. Data about PA invasive features were available in 7 studies with dichotomous-data and 2 studies with continuous-data (Table 4). In Galbraith plots for dichotomous-data studies (Figure 2A), all points fell within the appointed area, which indicates that there were no statistical heterogeneities across all the studies (Q = 3.44, d.f. = 6, I^2^=0.0%). As shown in Figure 3A, using a random-effect model, pooled OR indicates correlations between survivin expressions and invasiveness (OR 6.226, 95% CI 3.970, 9.765; P<0.001). These suggested that high survivin expression in postoperative PA tissues can predict a high invasive tendency. Conversely, in Galbraith plots for the continuous-data studies (Figure 2B), there are points that fall outside the area, suggesting relatively high heterogeneities (Q = 33.72, d.f. = 1, I^2^ = 97.0%). As shown in Figure 3B, also using a random-effect model, the SMDs of the 2 studies didn't suggest that survivin expression level was associated with the PA invasive tendency (SMD −5.043, 95% CI −10.965, 0.878; P = 0.095). All the meta-analysis results were shown in Table 6.

**Table 6:** The results of the meta-analysis for association between survivin and pituitary adenoma's invasiveness

Data type	Study or subgroup	Publishing year	Non-invasive groups			Invasive groups			% Weight	OR	95% CI
			Negative	Positive	Total	Negative	Positive	Total			
	Shi QH et al.	2005	25	7	32	15	24	39	18.10	5.714	[1.984, 16.455]
	Zhang ZQ et al.	2006	11	10	21	5	22	27	12.08	4.84	[1.326, 17.666]
	Zhou J. et al.	2008	15	5	20	8	21	29	12.00	7.875	[2.148, 28.868]
	Wang CL et al.	2008	17	14	31	10	41	51	20.70	4.979	[1.852, 13.386]
Dichotomous	He XL. et al.	2008	23	5	28	4	11	15	9.02	12.65	[2.827, 56.597]
	Jankowska A et al.	2009	16	11	27	4	35	39	12.20	12.727	[3.510, 46.152]
	Zhang YX et al.	2010	27	10	37	9	12	21	15.90	3.6	[1.165, 11.127]
	Integrated/pooled		134	62	196	55	166	221	100.00	6.226	[3.970, 9.765]
	Heterogeneity chi-squared = 3.44 (d.f. = 6) p = 0.752I-squared (variation in OR attributable to heterogeneity) = 0.0%					Estimate of between-study variance Tau-squared = 0.0000Test of OR=1 : z= 7.97 p = 0.000					
	**Study or subgroup**	**Publishing year**	**Non-invasive groups**			**Invasive groups**			**% Weight**	**SMD**	**95% CI**
			**n1**	**mean1**	**sd1**	**n2**	**mean2**	**sd2**			
	Jankowska et al.	2008	7	5.5	0.225	15	6	0.25	50.62	−2.06	[−3.161, −0.959]
Continuous	Xiang et al.	2009	20	3.09	1.03	30	10.28	0.78	49.38	−8.102	[−9.819, −6.385]
	Integrated/pooled		27	-	-	45	-	-	100	−5.043	[−10.965, 0.878]
	Heterogeneity chi-squared = 33.72 (d.f. = 1) p = 0.000I-squared (variation in SMD attributable to heterogeneity) = 97.0%					Estimate of between-study variance Tau-squared = 17.7150Test of SMD=0 : z= 1.67 p = 0.095					

### Sensitivity analysis and publication bias

**Figure 4:**
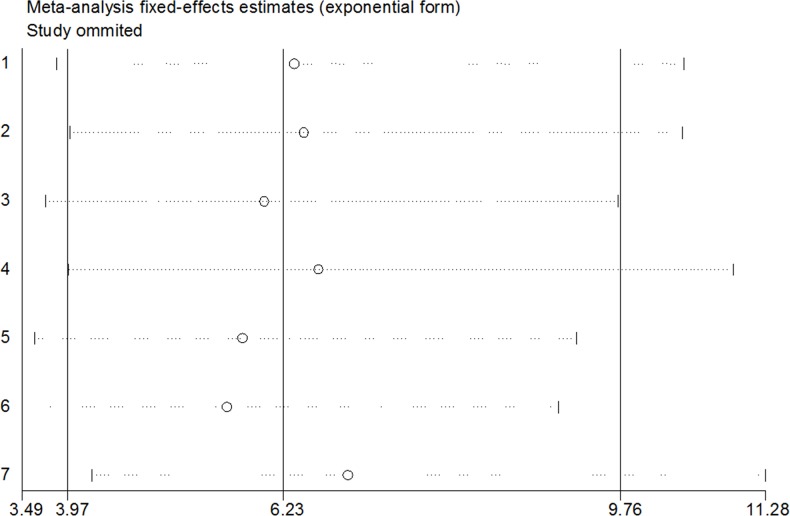
Sensitivity analyses of the seven dichotomous studies Results were computed by omitting each study in turn. Meta-analysis random-effect estimates (exponential form) were used. The two ends of the dotted lines represent the 95% CI.

**Figure 5:**
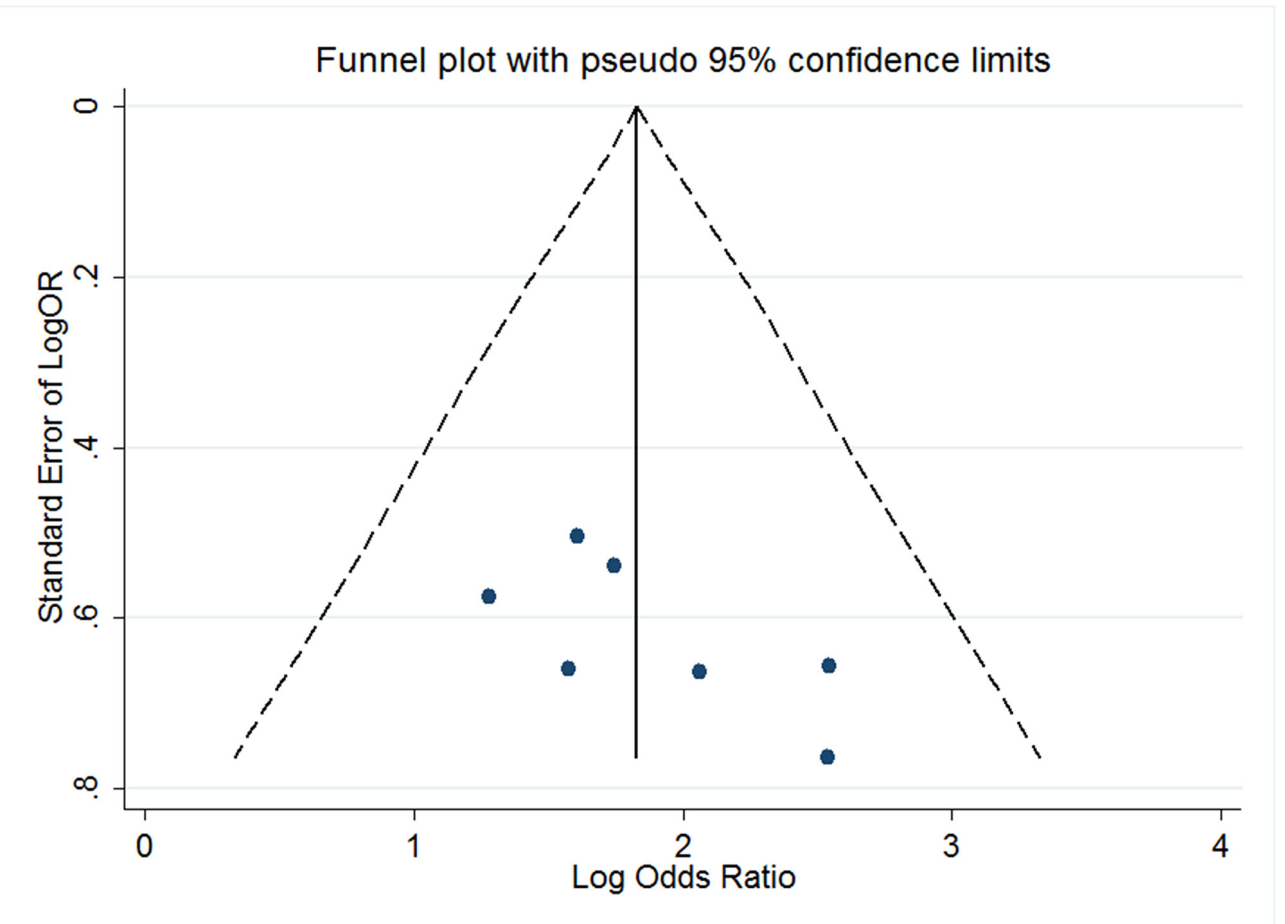
Funnel plot designed to visualize a potential publication bias

Sensitivity-analyses were conducted to calculate the influences of each individual study on the integrated ORs or SMDs. The results of the analysis suggested that no individual studies significantly affected the pooled OR of survivin and the invasiveness, indicating a statistically robust result (Figure 4). Because there were only two included studies with continuous data, a sensitivity analysis was not undertaken. Using Begg's and Egger's tests, no publication biases were detected among the 7 studies with dichotomous data (P=0.098, 95% CI-0.947, 8.118). In addition, a funnel plot revealed that the overall distribution was symmetric (Figure 5), indicating that there was no significant publication bias.

### Verification results

The expression levels of survivin in all invasive PA tissues and non-invasive PA tissues were shown in Table 7. A significant positive correlation was observed between survivin expression and the PA's histopathological invasiveness. The invasive PA tissues had much higher survivin expression than non-invasive PA tissues (P < 0.01).

**Table 7:** The expression of survivin in invasive and non-invasive pituitary adenomas

Type	n	-	+	++	+++	++++	Positive rate
**Invasive**	36	19	7	4	3	3	47.22%
**Non-invasive**	31	20	7	4	0	0	35.48%

## DISCUSSION

The involvements of survivin in tumorigenesis have been documented, mainly focusing on apoptosis inhibition and cell proliferation control. In recent years, it has been found that survivin gene knockdown could decrease the malignancies of gliomas [11]. Because of its vital roles in tumor cells’ biology, survivin is thus considered to be a potential bio-marker for prognosis and a potential bio-target for treatment. But it's still not clear enough whether survivin also plays important roles in PA invasiveness. To date, the impact of survivin on IPA is controversial.

We explored the survivin expressions in 9 studies and its associations with PA's invasiveness in 489 patients. We conducted quality assessments of the included literatures via browsing and scoring every article according to the methodological quality assessment scale (Table 2). Overall, the scores of the nine studies in terms of design, method, generalizability and results analysis remains fine, suggesting a relatively superior quality. The analysis for the 7 dichotomous-data studies indicated that survivin over-expression was closely correlated with the invasive tendency (OR 6.226, 95% CI 3.970, 9.765; P<0.001), but the analysis for the 2 continuous-data studies did not reveal any significant associations (SMD −5.043, 95% CI-10.965, 0.878; P = 0.095). However, the latter 2 studies only make up a small part, therefore we believe that the present analysis still indicated a relatively strong association between survivin overexpression and IPA. Because included data are not big enough, on this occasion, more evidence-based studies are necessary to further support this conclusion. The results of the present meta-analysis are consistent with the findings of our own study. In this study, we obtained tissue samples of 36 invasive PAs and 31 non-invasive PAs. The tumor samples were diagnosed by two pathologists who were blinded to the patient data. We adopted immunohistochemical methods to measure the expression of survivin in PA tissues. The results showed that the expression of survivin was significantly lower in non-invasive PA tissues than in invasive tissues (p < 0.05, Table 7). Our results further confirmed this association.

Galbraith plots, I^2^ tests and Cochran's Q-tests were all conducted to estimate the heterogeneities among the included literatures. A P value > 0.05 and/or I^2^ < 50% indicate homogeneity. The fixed versus random effects model should be based on sampling population and not purely on heterogeneity considerations. It seems likely that there are considerable phenotypic variations between populations in the different studies, so we use the DerSimonian and Laird random-effects model no matter the I^2^ was higher than 50% or lower than 50%. As for limitations, first, most included studies were done in Asian populations, which may have led to selection bias. Second, the criteria or methods adopted to measure survivin expressions weren't consistent. Third, to a large extent, IHC results depend largely on methodological factors such as primary and secondary antibody titer. It was difficult to perform subgroup-analyses involving different antibodies to evaluate possible method biases regarding the final incorporated results. Moreover, some studies did not present complete data, though this might not have affected the bias.

Publication-bias is another concern in meta-analyses [12]. Most articles tend to report positive outcomes, while the articles with negative findings are usually rejected. In our study, neither Begger's and Egger's P value tests nor funnel-plots suggested publication-bias. However, since the languages used in the included articles in this meta-analysis was mainly Chinese and English, there may exist potential publication-bias.

## CONCLUSION

This meta-analysis indicates that survivin is closely associated with high invasive tendency of PAs and it may be an important prognostic factor. In addition, survivin detection might facilitate new insights into cancer grades’ prediction and early treatment-regimens of patients undergoing operational resection. However, not all the studies have a perfectly desirable scientific quality and there actually exist inconsistent results between the dichotomous studies and continuous studies, so to obtain a more accurate conclusion, more data obtained using medical big data are necessary to support this correlation.

## MATERIALS AND METHODS

### Search strategy

A global search strategy was systematically applied among five databases including Cochrane Library, Embase, PubMed, Web of Science, and Chinese National Knowledge Infrastructure (CNKI) up to June 18th, 2017. No language restriction was set. Detailed searching strategies were in Table 1. For PubMed, we adopted “(“pituitary neoplasms”[MeSH Terms] OR (“pituitary”[All Fields] AND “neoplasms”[All Fields]) OR “pituitary neoplasms”[All Fields] OR (“pituitary”[All Fields] AND “adenoma”[All Fields]) OR “pituitary adenoma”[All Fields]) AND survivin [All Fields]” as the search strategy and got 26 articles. To find out other potentially eligible studies, we also reviewed related references, but got no new studies. Meanwhile, we tried to contact authors to identify additional studies and request missing data (firstly through email, if no responses, we would try to contact the authors by telephone). Together, we identified 151 articles.

### Study selection

Two reviewers selected studies independently. Disagreements were settled by discussion or solved by a third reviewer. Inclusion criteria were as follows: (1) a diagnosis of PA confirmed by a pathologist; (2) PA invasiveness as a primary outcome of the study (note: generally, invasion/invasiveness is defined as an infiltration and often destruction of parasellar tissues, including the leptomeninges, subarachnoid space, paranasal sinuses, cranial nerves, cavernous venous sinuses, bone and dura. The definition of invasiveness and non-invasiveness is usually made using radiological evidences of invasion on MIR and/or by intra-operative exploration of parasellar tissues and sellar walls; (3) a survivin expression model established by real-time quantitative polymerase chain reaction (qPCR), reverse transcription PCR (RT-PCR), immunohistochemistry (IHC) or other reliable molecular-biological technological means; (4) mean values with SD, ORs with 95% CI between survivin expressions and the invasiveness could be calculated according to the tables or figures in the article (for example, if the study only presented primary study data instead of a SD value, we can calculate the needy data ourselves), directly obtained, or by contacting the authors.

### Data extraction

The following data were extracted independently by two investigators from the included studies using a specially designed table: first author name, publication year, nationality, pathology, study methods, invasive behaviors, patient numbers, mean age and the positive percentage of survivin expressions in the tumor specimen. Disagreements between the two investigators would be settled by a third reviewer.

### Quality assessment

In accordance with the methodological quality assessment scale (see Table 2), two investigators independently estimated the qualities of the included literatures. Disagreement would be solved by discussion. In this methodological quality assessment scale, five items, including quality control of sample size, source of controls, genotyping methods, cases representativeness and HWE were checked carefully. The quality scores range between 0 ∼ 10, and the higher the score is, the better the qualities are.

### Data synthesis and analysis

The meta-analysis was in accord with the PRISMA checklists and guidelines (it can be accessed through http://www.prisma-statement.org/). All statistical analysis was performed using STATA 12.0 (StataCorp LP, College Station, TX, USA). Differences were expressed as ORs with 95% CIs or SMDs with SD. The I^2^ tests, Cochran's Q-tests, and Galbraith figures were used to estimate the heterogeneities between studies [13, 14]. In the Galbraith plot, circles that are all distributed within the region bounded by the upper line and lower line indicates that there is no statistical heterogeneities. Heterogeneities were also considered significant if P < 0.05 (Q statistic) [15]. I^2^ values of 75%, 50% and 25% refer to high, moderate and low heterogeneities, respectively. The fixed versus random effects model should be based on sampling population and not purely on heterogeneity considerations. It seems likely that there are considerable phenotypic variations between populations in the different studies, so we use the DerSimonian and Laird random-effects model both studies with dichotomous data and studies with continues data no matter the I^2^ was higher than 50% or lower than 50% [15]. Theoretically, stratified analysis based on ethnicities and methods should be performed to avoid or decrease possible bias interference. However, in all the included 9 studies, only one study was conducted on Caucasian population and all the other 8 studies were on Asian population, thus the stratified analysis based on ethnicities makes little sense. Similarly, for the survivin test methods or measurement technique, only 1 study used RT-PCR, while all the other 8 studies used HIC, so the thus the stratified analysis based on methods was also waived. In the future study, when there are more included studies, stratified analyses based on ethnicities and methods are highly recommended to perform.

Using the one-at-a-time statistical approach (remove one individual literature at a time and repeat the analysis process), we conducted a sensitivity-analysis to estimate the final results’ stability. Funnel plots were drafted to assess potential publication-bias. If the plot seems symmetric, it's reasonable to say there is no publication-bias [16]. The Egger linear regression tests were also performed (P < 0.05 suggests publication-bias) [17]. Because only 9 studies were included, we did not perform the meta-regression analysis.

### Further investigation and confirmation

#### Patients and tissue samples

We included and analyzed 67 surgically resected PA samples. Among these patients, there were 30 men and 37 women. Their ages ranged from 21 to 59 years old. Through radiological features on pre-surgical MIR and intra-operative impressions, the 67 samples were divided into 36 invasive ones and 31 age- and gender-matched non-invasive ones. All of these tissues were fixed in 10% formalin and embedded in paraffin. No patients had received preoperative radiotherapy, chemotherapy or any other biological therapies.

### Immunohistochemistry for survivin

Primary rabbit monoclonal antibodies to human survivin and secondary anti-rabbit antibody were used. the immnunohistochemical staining procedure was conducted based on the Cell Signaling Technology protocols. We semiquantitatively calculated the survivin immunoreactivities according to the staining-intensity. The percentage of positive adenoma cells were blindly evaluated in 5 areas at ×400. Standards to evaluate the intensity of survivin staining: negative (−), no expression or positive cells was 0%; +, light yellow or ≤10%; ++, brown yellow or 11%∼50%; +++, brown or 51%∼75%; ++++, dark brown or >75%.

### Statistical analysis

All computations were carried out using the software of SPSS version 13.0 for Windows (SPSS Inc., IL, USA). None parameter rank and summing tests were performed. Differences were considered statistically significant when P was less than 0.05.

## Abbreviations

PA: pituitary adenoma
IPA: invasive pituitary adenoma
CNKI: China National Knowledge Internet
OR: odds ratio
SMD: standard mean difference
CI: confidence interval
IHC: immunohistochemistry
RT-PCR: reverse transcription polymerase chain reaction
qPCR: quantitative polymerase chain reaction
SD: standard deviation
